# Minimizing endpoint variability through reinforcement learning during reaching movements involving shoulder, elbow and wrist

**DOI:** 10.1371/journal.pone.0180803

**Published:** 2017-07-18

**Authors:** David Marc Anton Mehler, Alexandra Reichenbach, Julius Klein, Jörn Diedrichsen

**Affiliations:** 1 Institute of Cognitive Neuroscience, University College London, London, United Kingdom; 2 Department of Psychiatry and Psychotherapy, University of Münster, Münster, Germany; 3 Cardiff University Brain Research Imaging Centre (CUBRIC), School of Psychology, Cardiff University, United Kingdom; 4 Department for Computer Science, Heilbronn University, Heilbronn, Germany; 5 Tecnalia Research and Innovation, Donostia-San Sebastián, Spain; 6 Brain and Mind Institute, Western University, London, Canada; VU University Amsterdam, NETHERLANDS

## Abstract

Reaching movements are comprised of the coordinated action across multiple joints. The human skeleton is redundant for this task because different joint configurations can lead to the same endpoint in space. How do people learn to use combinations of joints that maximize success in goal-directed motor tasks? To answer this question, we used a 3-degree-of-freedom manipulandum to measure shoulder, elbow and wrist joint movements during reaching in a plane. We tested whether a shift in the relative contribution of the wrist and elbow joints to a reaching movement could be learned by an implicit reinforcement regime. Unknown to the participants, we decreased the task success for certain joint configurations (wrist flexion or extension, respectively) by adding random variability to the endpoint feedback. In return, the opposite wrist postures were rewarded in the two experimental groups (flexion and extension group). We found that the joint configuration slowly shifted towards movements that provided more control over the endpoint and hence higher task success. While the overall learning was significant, only the group that was guided to extend the wrist joint more during the movement showed substantial learning. Importantly, all changes in movement pattern occurred independent of conscious awareness of the experimental manipulation. These findings suggest that the motor system is generally sensitive to its output variability and can optimize joint-space solutions that minimize task-relevant output variability. We discuss biomechanical biases (e.g. joint’s range of movement) that could impose hurdles to the learning process.

## Introduction

Learning a new motor skill often requires the coordinated action across several joints. The biomechanics of the human body equip us with abundant degrees of freedom, meaning that many different movements in joint space achieve the same task goal. How the brain picks one of the options for executing a motor action remains an important question in motor neuroscience [1]. When performing a backhand stroke in tennis, for example, different combinations of joint movement in the trunk, shoulder, elbow and wrist yield a successful hit. However, there will be some joint configurations that allow for more control over the racket, and therewith reduce the variability of the returning ball trajectory and increase the success of achieving the desired action [2]. The many years of training required to become a motor expert are, to some degree, spent on acquiring the optimal movement solutions in joint space. What are the learning mechanisms that underlie this process?

**Fig 1:**
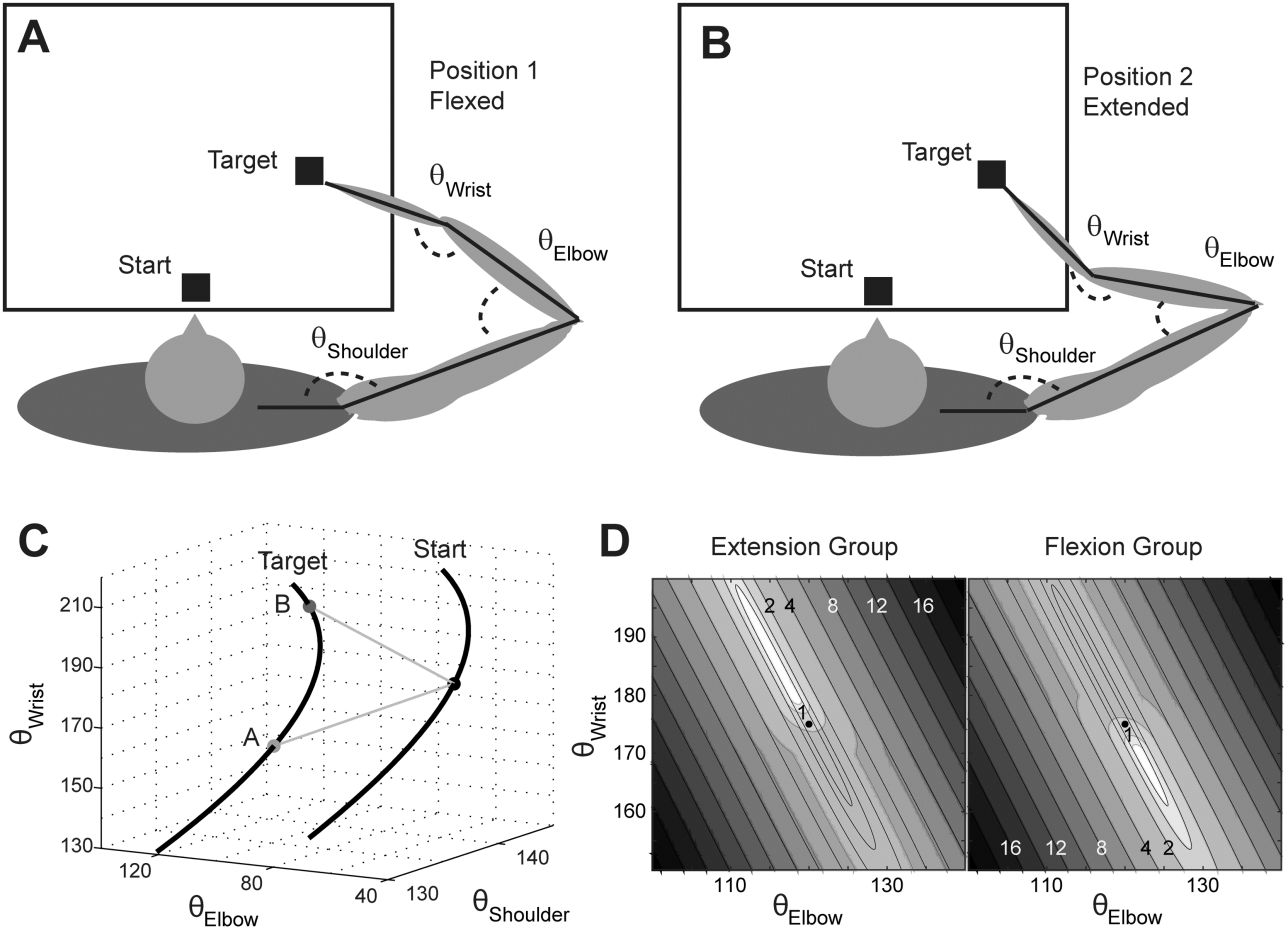
Task geometry. (A, B) Schematic illustration of the setup. (A) Top view on a participant in the redundant task environment with an exemplary end position demonstrating a flexed wrist joint configuration (***θ***_***wrist***_ = 160°). (B) Exemplary end position demonstrating an extended wrist joint configuration (***θ***_***wrist***_ = 210°). (C) Start and target positions defined a solution manifold in joint space (black lines). For any joint configuration along this line the effector endpoint, i.e. the fingertip, remained at the same position. The black dot on the start line represents the enforced start configuration (***θ***_***wrist***_ = 175°) and the two connection lines represent two possible joint trajectories to the flexed (bottom) and extended (top) end configurations from panels A and B. (D) Illustration of the mean absolute visual reaching error in cm (distance between visual target and cursor feedback) as a function of wrist and elbow angles for a fixed shoulder angle. The visual reaching error was here simulated for each wrist angle by drawing visual noise 100.000 times from a standard distribution with zero mean and SD as a function of wrist angle (see Eq 2). The black dot indicates the average elbow and wrist angle of the participant during the baseline block for trials that were on target. In the extension group, increased wrist flexion is penalized by added error; in the flexion group, wrist extension is penalized. The thin black lines illustrate the actual reaching error without the visual noise (absolute distance between visual target and actual hand position). Note that the solution manifold bends beneath the depicted plane for wrist and elbow angles further away from the baseline angles giving the error zones an ellipsoid instead of striped appearance.

To investigate this learning process, we used a situation with one redundant degree of freedom. Participants made planar reaching movements with the combined motion of the shoulder, elbow and wrist joints. Therefore, many different joint configurations led to the same movement end point (Fig 1A and 1B). In joint space, these equivalent solutions form a 1-dimensional manifold (left black line in Fig 1C), whose non-linear shape is determined by the geometry of the arm. Because variation along this manifold is task-irrelevant (i.e., does not change the endpoint) it does not need to be corrected for [3]. Therefore, this subspace is also called the uncontrolled manifold (UCM) [4–6] or solution manifold [7]. Typically, it is observed that variability along this manifold is larger than the variability orthogonal to the manifold [5,6].

Error-based learning enables the motor system to correct for deviations away from the reaching target in the task-relevant dimension, i.e. it corrects towards the solution manifold. This learning mechanism utilizes the endpoint deviation to directly update the next movement [8–10]. During this process, the motor system needs to convert visual errors perceived in three-dimensional world coordinates into a movement correction performed in joint space and thus requires knowledge of the geometry of the motor plant. The mechanism has therefore been characterized as model-based learning [11]. It is likely driven by sensory prediction errors and depends heavily on the integrity of the cerebellum [8,9,12]. Error-based learning therefore quickly reduces the average error by bringing the joint space solution onto the solution manifold [4,13].

The movement strategies along the solution manifold show no systematic error or bias. However, among these possibilities may be solutions that are less effortful [14–16], dynamically more stable [17], or more error-tolerant leading to reduced output variability [4]. In such situations, error-based learning fails to inform the motor system which of the many solutions along the solution manifold to pick. Thus, the motor system requires other learning mechanisms to identify preferable solutions. One candidate is model-free reinforcement learning. This mechanism has been studied in various domains of neuroscience including decision making [18,19], perceptual learning [20,21], and more recently in motor control [7,22,23]. In its simplest form, reinforcement learning requires only a signal that informs whether a movement was correct or not [24]. More sophisticated forms of reinforcement signals may also provide graded feedback about how successful the movement was [25]. Importantly, however, reinforcement signals do not provide directional information as to how to change the motor command [11]. Thus, the learning mechanism needs to rely on active exploration along the solution manifold to determine the movements that yield increases in success [23,26].

A recent study investigated reinforcement learning in a series of experiments using a redundant two-dimensional reaching task [23]. The main finding was that explicit binary feedback about task success or failure led to very fast learning when participants were aware of the dimension along which they had to change their behavior [see also 28]. Learning was in general absent when participants were not explicitly aware of this direction. The authors found only significant learning when reward probability was manipulated by adding noise to the cursor feedback, which gave participants the impression of higher variability and lower controllability for certain movements.

In most natural tasks, redundancy is a consequence of the human arm configuration. When we try to improve motor skills, we are often not aware of the exact dimension in joint-space that can help reduce variability—unless we have a coach that provides proximal feedback, such as “perform the swing out of the wrist”. To capture the challenge of a natural reinforcement learning problem, we therefore designed a task in which participants had to reach to targets on a plane through a coordination of shoulder, elbow and wrist movements (Fig 1A and 1B). In contrast to most previous reinforcement studies [25,27,28] we did not provide explicit instructions about the critical learning dimension to participants. The main novelty in the current design was that we only implicitly reinforced a specific joint configuration (more flexion or extension in the wrist joint relative to baseline behavior). This advancement was possible by using a novel robotic manipulandum capable of measuring and controlling the three main joints of the arm (shoulder, elbow and wrist) [29], which ensured the same starting position and joint configuration for the beginning of each trial. To encourage specific movement solutions, we added noise to the visual endpoint feedback, similarly to the implicit feedback condition used by Manley et al. [23]. Participants could only avoid this injected variability if they adopted a new arm configuration, depending on the experimental group either more flexion or extension in the wrist joint. Hence, this design exploited the natural redundancy of the arm in joint space for reaching a specified position in endpoint space and thus mimics the challenge of finding a favorable joint-space movement for a new motor skill without explicit knowledge of the dimension along which variability changed. We also investigated whether the amount of this learning mechanism correlated with the behavioral variability at baseline, thereby testing the hypothesis that increased exploration is related to more reinforcement learning [26].

## Materials and methods

### Participants

Forty-two right-handed participants [30] without any history of neurological or psychiatric diseases were recruited from an internal experiment database. We tested two experimental groups (*flexion group*, n = 13; *extension group*, n = 13) and two control groups (*low-noise control group*, n = 9; *high-noise control group*, n = 7). Participants’ age ranged between 18 and 36 years and 75% of participants were male, without significant differences between groups. All participants provided written informed consent prior to testing and were paid as compensation for their time expense. They were naïve to the purpose of the experiment and debriefed after the experimental sessions. The research ethics committee of University College London approved all experimental and consenting procedures. Data of one participant from the e*xtension group* was excluded before analysis because the participant changed the body position in the setup and thereby gained direct vision of the workspace (see Apparatus and Stimuli).

### Apparatus and stimuli

Participants were seated comfortably in front of a virtual environment setup, leaning slightly forward with their chest and forehead supported by a chest- and forehead rest, respectively. The experimental chair was fixed in a comfortable position, avoiding changes of participant’s position and especially rotation movements around the body’s yaw axis. Customized chest belts fixed participants’ trunk orientation and shoulder position throughout the experimental session. Participants made 15cm straight reaching movements in a horizontal plane at shoulder level (Fig 1) while their right hand was attached to a robotic manipulandum [29]. The robotic manipulandum allowed for free planar movement. The participant’s arm was supported by a lightweight hand and forearm rest, and the robot was equipped with an actuated joint that allowed flexion and extension of the wrist joint. The length of the robotic wrist from the rotation axis of the wrist joint to the tip of the finger was 16 cm. Participants could achieve movements of the endpoint in the planar workspace through wrist, elbow and shoulder movements, which provided 1 degree of redundancy in control. Direct vision of wrist and elbow was prevented by a mirror mounted horizontally above the manipulandum. The mirror provided the view on the visual scene from a top-mounted LCD monitor (update rate 60 Hz). The apparatus was adjusted such that the visual and haptic scene were congruent.

### Calibration and kinematics

The 3-degree-of-freedom (3-DoF) robotic manipulandum used in this study provided position data of the wrist joint and the absolute orientation of the wrist handle in robot space. We were interested in quantifying changes in angles between limb segments, i.e. the relative joint angles θ _shoulder_, θ _elbow_, and θ _wrist_ (cf. Fig 1A and 1B). Together with the position of the shoulder joint (pos _shoulder_), these three relative joint angles determine the position of the finger tip (i.e. endpos) for a planar 3-DoF arm:

$$\begin{array}{ll} endpos= & \;\left[\begin{array}{c} -\text{cos}\left(\theta_{shoulder}\right)\\ sin\left(\theta_{shoulder}\right) \end{array}\right]*L1+\;\left[\begin{array}{c} \text{cos}\left(\theta_{shoulder}+\theta_{elbow}\right)\\ -\text{sin}\left(\theta_{shoulder}+\theta_{elbow}\right) \end{array}\right]*L2\\ & +\left[\begin{array}{c} -\text{cos}\left(\theta_{shoulder}+\theta_{elbow}+\theta_{wrist}\right)\\ \text{sin}\left(\theta_{shoulder}+\theta_{elbow}+\theta_{wrist}\right) \end{array}\right]*L3+pos_{shoulder} \end{array}$$ (1)

In Eq 1, L3 denotes the length of the wrist handle, and L1 and L2 denote the length of the upper and lower arm, respectively. L3 was predetermined with 16cm from the setup. pos _shoulder_, L1 and L2 were estimated for each participant with a calibration procedure at the beginning of the session. To establish the stability of our calibration and testing procedure, we assessed the within-session retest reliability of the estimated measures by testing an additional group of participants (*evaluation group*, n = 14, 10 males, 21–29 years). The retest reliability between the first and second set of calibration was consistently high across all four measurements (*r*_Shoulder-X_ = 0.83; *p* < 0.001; *r*_Shoulder-Y_ = 0.75; *p* < 0.001; *r*_L1_ = 0.82, *p* = 0.001; *r*_L2_ = 0.92; *p* < 0.001).

### Trial procedure

A trial started with the presentation of the start box (unfilled white square, 1.0cm size, at body midline, Fig 1A and 1B). To achieve a constant configuration of the arm at the start of a trial, we provided veridical feedback about the wrist joint position (filled white hexagon, 0.5 cm diameter) and the finger endpoint position (cursor, unfilled white circle, 0.5 cm diameter) connected by a white line (16 cm length).

Participants had to move the cursor into the start box and align their wrist to a fixed pink template line connected to the start box, which indicated the required wrist angle (175 ± 2°, i.e. slightly flexed). The starting position and wrist angle were identical for participants across all experimental and control groups. After holding this position for 800ms, a target box (unfilled white square, 1 cm size) located 15 cm diagonal to the right of the start box (Fig 1A and 1B) appeared, indicating the start of the trial. Simultaneously, the start box and the cursor disappeared to eliminate visual feedback about the cursor position until the end of the trial. Participants were instructed to move their unseen fingertip into the target box quickly and accurately performing whole arm movements. Importantly, participants were not explicitly instructed that some solutions would be associated with a higher chance to score points than others. The trial ended when the tangential endpoint velocity remained below 3.5 cm/s for 200ms. With trial end, the cursor was re-introduced shortly to provide visual feedback, including added noise if applicable. Subsequently, the cursor disappeared again to mask the potential offset between given and veridical feedback and the robotic arm guided the participants back to the start position where veridical feedback reappeared to allow alignment of the wrist within the starting box.

A trial was reported back to the participant as valid and increased the score when reaching time was shorter than 700ms and maximum cursor velocity ranged between 45 and 100 cm/s (the criteria used in data analysis were more liberal; see section *Data Analysis*). Only valid trials with visual end point (i.e. the actual fingertip plus the noise) accuracy of at least 1 cm were rewarded with a visual target “explosion” and a point, with the cumulative point-score presented continuously on the screen. Additionally, invalid, valid and point scoring trials were indicated by a color scheme applied to the cursor at the end of each trial. To keep participants motivated, we displayed an artificial high-score list at the end of each experimental block, in which participants were randomly ranked on the top three places amongst virtual competitors.

### Experimental conditions

The purpose of the experiment was to test whether participants would learn to reach into arm configurations that avoided large endpoint variability. We characterized the position along the solution manifold using the wrist angle, which uniquely determined the whole arm configuration, assuming the fingertip is at the target (Fig 1C). To reinforce end postures with more extension or flexion of the wrist relative to a baseline posture, we added stochastic noise to the cursor at the endpoint when movements were performed into joint configurations that exhibited a behavior opposite to the rewarded one. This means, when wrist extension was reinforced (cf. position B in Fig 1C), wrist flexion was penalized by adding noise to the shown endpoint position. The noise was normally distributed with the standard deviation depending linearly on the difference between reinforced and actual wrist angle (for details see Eq 2). Thus, movements finishing in joint configurations opposing the goal configuration yielded on average a larger absolute visual error than movements in the direction of the goal configuration (Fig 1D), therefore decreasing the chance of a successful trial. As a consequence, we expected participants to prefer solutions with low or no added endpoint variability, i.e. with low or without injected noise. Since no visual feedback about the hand was provided during the movement and during the return to the start location, participants did in general not notice the manipulation of their visual endpoint feedback.

First, a baseline block (B0) of 70 trials was recorded to determine the average wrist angle of each participant when landing on the target. This value served as reference for the midpoint of the noise gradient during the subsequent learning blocks (B1-B8). To prevent an abrupt onset of visual noise in the learning blocks, which could make participants suspicious of the truthfulness of the visual feedback, we added noise with a constant standard deviation (SD) of 1cm already during baseline. During the experimental blocks (B1-B8) participants were exposed to a noise gradient, based on which they could decrease the SD by increased flexion or extension in their wrist angle at the end of the movement (Fig 1D). If the relative wrist angle at target position was the same as the reference value during the baseline block, the noise added to the visual feedback was drawn from a normal distribution with 2cm SD. From -5° to +5° around the reference angle, the SD increased or decreased linearly (see Eq 2). Beyond the ±5° boundary, the SD would not increase or decrease further.

SD = 2cm+(k) 2cm/5° * (current relative wrist angle − reference angle from B0) (2)

In Eq 2, k is either -1 if the behavioral change was in the expected direction, +1 otherwise. More specifically, participants in the *flexion group* could decrease the SD of noise to 0cm by flexing the wrist joint 5° or more than baseline and were penalized with noise with up to 4cm SD for wrist extension. This yielded mean absolute injected noise in end point space for the two experimental groups as depicted in Fig 1D. Correspondingly, participants in the *extension group* were penalized for flexing the wrist joint, but could decrease the SD to 0cm by extending their wrist 5° or more than baseline. To test whether the mere presence of noise (without a gradient) influenced participant’s exploration and hence also learning behavior, we tested two control groups. The *low-noise control group* always received veridical feedback without added noise, whereas the *high-noise control group* experienced added noise with a constant SD of 2cm, i.e. the added noise was independent of the wrist angle in both control groups.

### Session procedure

A session started with several training blocks to accustom participants with the setup and task, and to produce stable baseline task performance. First, they received veridical visual feedback about the cursor position during the movement until at least 75% of trials were valid. Subsequently, participants received one training block without visual feedback of the cursor position during the movement with the same noise on the visual endpoint feedback as in the baseline block (constant SD of 1cm). Afterwards, participants were informed that the experiment began. The first experimental block constituted the baseline (B0) for determining the reference wrist angle, followed by 8 learning blocks (B1-B8). Points scored during all 9 experimental blocks counted towards a bonus paid at the end of the experiment. After the experiment, participants were systematically interviewed and debriefed to determine whether they had become aware of the critical task dimension to control reaching accuracy. We first let participants report freely any strategy that they may have used during the task to maximize their score. Next, we told participants that there was a hidden dimension that had influenced task success and asked them to guess which dimension that was. Finally, we revealed that the relative wrist angle was the critical dimension and tested for implicit knowledge with a two-alternative forced choice (2AFC) question asking whether they thought they were in the extension or flexion group. Participants who mentioned or indicated the critical dimension correctly during first or second question were classified as *aware*.

### Data analysis

The relative joint angles for wrist, elbow and shoulder were calculated based on the arm model, the position of the robotic wrist joint, and the orientation of the wrist handle in the workspace using custom-written MATLAB routines (The MathWorks, Natick, MA, USA). The testing performance criteria were overly strict to encourage rapid reaching movements and would have led to an exclusion of ~7% of trials in both experimental groups. However, many of these trials were still relevant for the data analysis. Therefore, invalid trials were identified by using less strict criteria than during the testing. Movement-time (MT) threshold was relaxed to 800ms and maximum endpoint velocity to a range between 35 and 100 cm/s. These criteria led to an exclusion of 2.7% of all trials. Start and end time points of movements were defined as the velocity exceeding or falling below 2.5 cm/s for at least 40ms. To quantify learning effects, we calculated the mean change in the relative wrist angle with respect to baseline for each block (Δ Wrist). To quantify changes in injected noise, we calculated the mean absolute injected noise, i.e. the distance between the shown cursor position and the tracked cursor position in endpoint space for each block. To quantify reward rates, we calculated the percentage (%age) of successful trials that were rewarded during the experiment for each block. All values reported are mean values across participants and the respective standard errors of the mean (SEM) unless stated otherwise. T-tests were conducted two-tailed unless described otherwise. *P* values < 0.05 were accepted as significant.

To test for the hypothesis that exploration relates to motor learning, we used the standard deviation (SD) of the wrist angle during the baseline block as a proxy for exploration in the task-relevant dimension for each participant [26] and correlated it with their respective Δ Wrist from the last block (B8) as a proxy for learning. On group level, outliers were detected with the Grubb’s test for outliers after normality had been established (Lilliefors test). Identified outliers were removed from the correlation analysis (one participant of the *flexion group* and one participant of the *extension group*). The analysis of exploration suggested that exploration related to learning in only one group. Thus, to test for differences between correlations for the *flexion group* and *extension group* a Fisher r-to-z transformation was applied.

## Results

### Joint space trajectories

**Fig 2:**
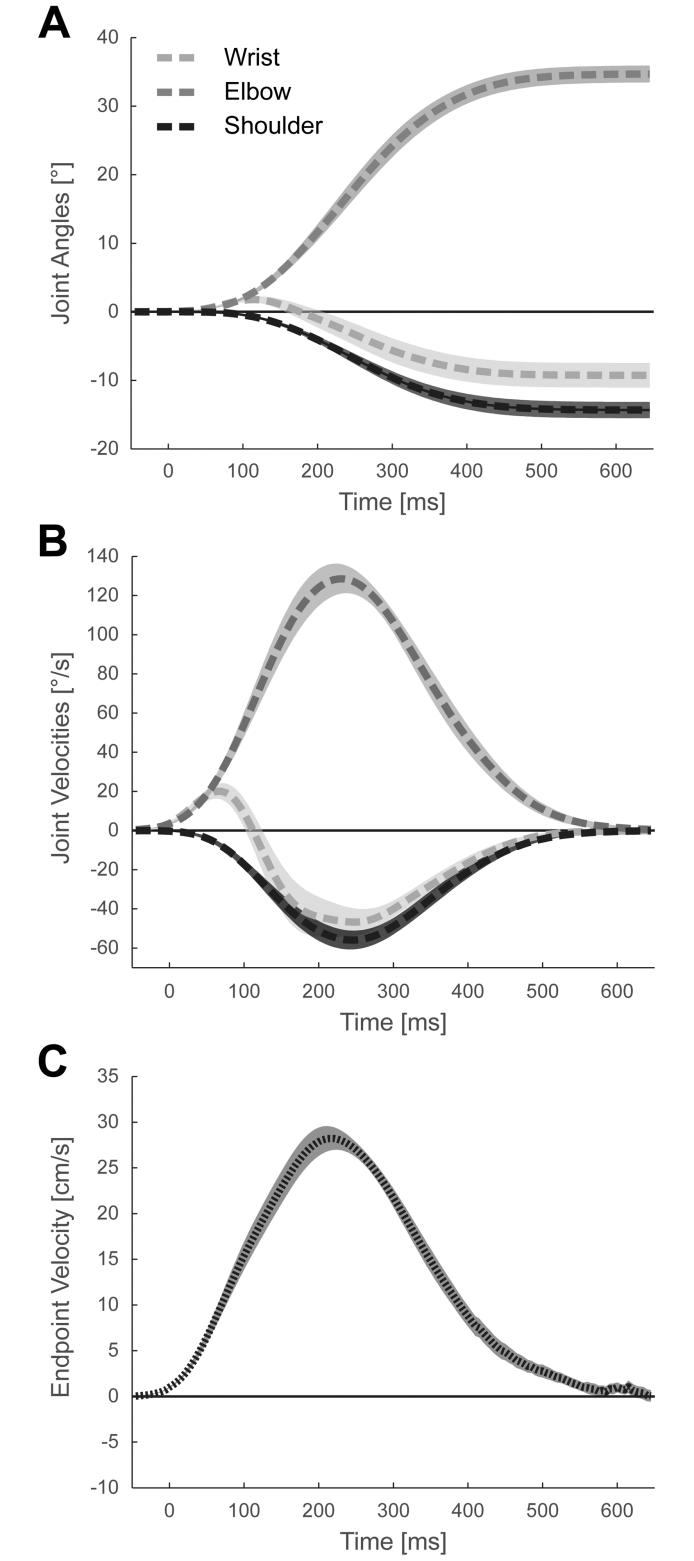
Basic movement kinematics. **(A)** Changes in the angle of shoulder, elbow and wrist joints during the baseline block, averaged across experimental groups. Angles are express relative to start configuration. (**B**) Angular velocities of shoulder, elbow and wrist joints. (**C**) Tangential velocity of the endpoint during baseline block. All data are averaged across experimental groups. Trajectories were aligned to the onset of movement (time = 0ms). Shaded areas denote the SEM across participants.

All participants made smooth controlled movements involving the wrist, elbow and shoulder joints. Visualizing the joint angles (Fig 2A) revealed that the movement was mostly accomplished by a combination of elbow extension (34.71 ± 1.23°) and shoulder flexion (-14.34 ± 1.18°), which is in line with earlier findings [31]. Interestingly, the wrist joint displayed a biphasic movement profile with an initial extension that peaked at 115 ± 15ms with 1.79 ± 0.41°, followed by a flexion (-9.29 ± 1.79°). Thus, the preferred movement strategy yielded a significantly flexed wrist joint at target compared to start position (*t*_*24*_ = 5.179; *p* < 0.001). As a consequence of the biphasic wrist movement, its velocity deviated from the bell shaped velocity profile observed in the elbow and shoulder joints and displayed a biphasic profile (Fig 2B) with an early extension peak (16.92 ± 3.69°/s) at 65 ± 5ms, followed by a flexion peak (-38.3 ± 6.43°/s) at 250 ±16ms. The elbow (122.21 ± 7.32°/s at 225 ± 9ms) and shoulder (-53.64 ± 4.3°/s at 240 ± 8ms) joints reached their peak velocities between the two peaks of the wrist joint. The tangential velocity of the finger-tip (Fig 2C), i.e. the controlled end-effector, peaked just before the elbow at 215 ± 7ms with 28.22 ± 1.32 cm/s.

To test for pre-existing behavioral differences between experimental groups, we compared movement kinematics during the baseline block (B0). First, we confirmed that both groups started trials at the desired wrist angle (*flexion group*: 174.75 ± 0.07°; *t*_*12*_ = 1.016; *p* = 0.330; *extension group*: 174.99 ± 0.12°; *t*_*11*_ = -0.015; *p* = 0.988). There was no evidence for a difference between groups (*t*_23_ = -1.789 *p* = .087). Second, we found no differences in wrist angle at target position (165.17 ± 2.24° vs. 166.01 ± 3.00°; t_23_ = -0.225; *p* = 0.824), confirming that both groups flexed the wrist joint at target compared to start position. Finally, reaction time (376.9 ± 36.3ms vs. 359.0 ± 36.2ms; *t*_23_ = 0.349; *p* = 0.731) and movement time (432.1 ± 15.4ms vs. 476.1 ± 26.4ms; *t*_23_ = -1.467; *p* = 0.156) were comparable between experimental groups. Thus, it is unlikely that pre-existing group differences in movement kinematics might explain any differences found in the learning blocks. However, it should be noted that the preferred naïve (baseline) reaching strategy of both groups was a relative wrist flexion.

### The motor system can implicitly learn a new joint-space trajectory

The main question of the experiment was whether participants could learn a new joint-space trajectory in the solution manifold to reduce variability and to optimize reward, without being aware of the experimental manipulation. Therefore, we excluded 5 participants (2 from the flexion group, 3 from the extension group) who reported the manipulated task dimension during the post-experiment interview (see Methods) and were thus classified as *aware participants*. The remaining group of 20 participants gave no indication of explicit awareness. This was supported by the group result of the final 2AFC question that was answered correctly only by 10 subjects, which constitutes chance level based on a binominal test (*p* = 1). This ratio suggests that this group had no explicit knowledge about the task critical dimension. Therefore, we performed the subsequent analyses based on this subsample of 9 participants for the extension and 11 participants for the flexion group.

**Fig 3:**
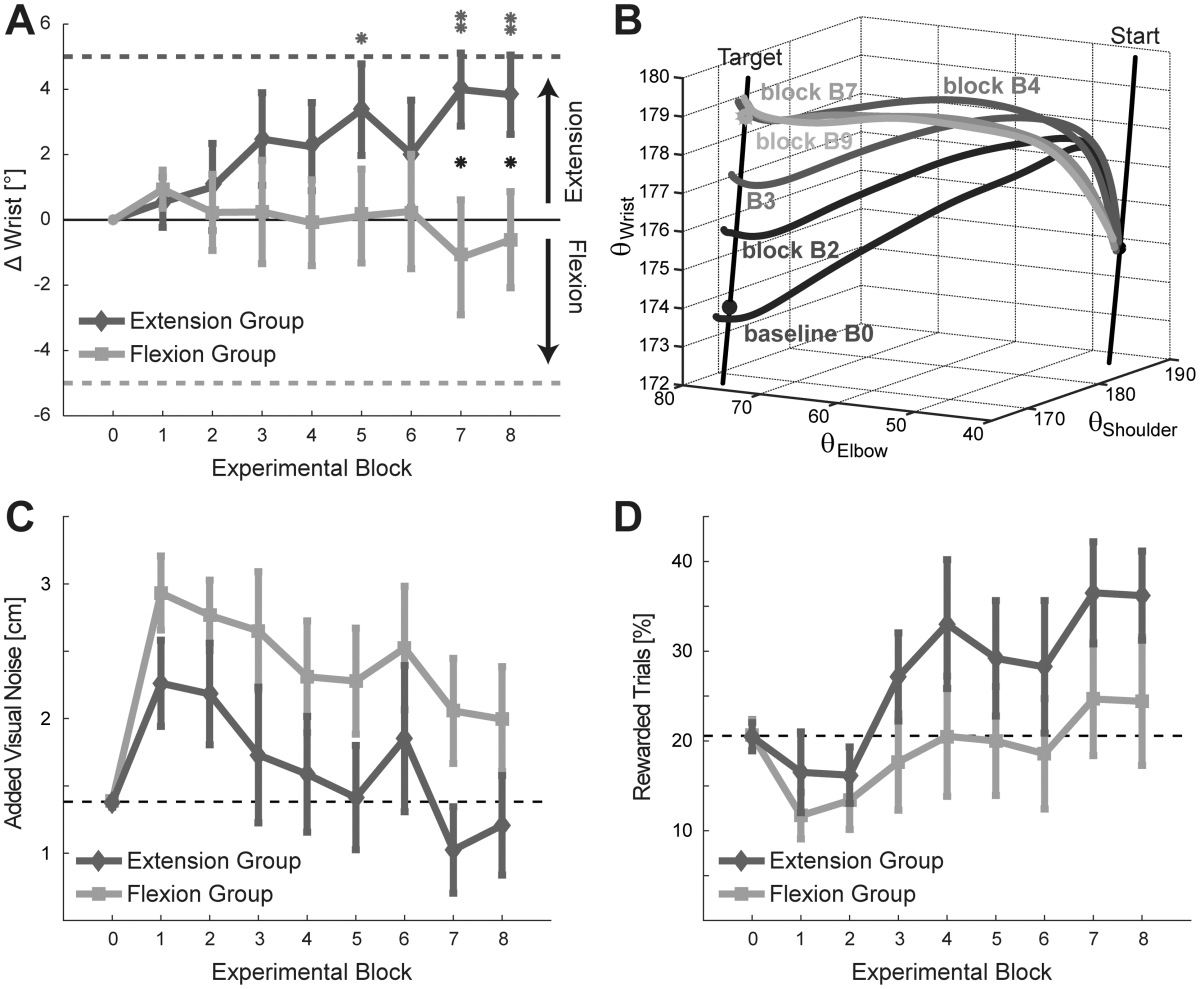
Learning of experimental groups. (A) Change in wrist movement over the time course of learning split by experimental group. The horizontal lines at ± 5° indicate the learning goals for each group. Error bars denote SEM across participants. T-tests for comparison each group mean vs. 0 are indicated in group color, between group comparisons in black: * *p* ≤ .05; ** *p* ≤ .01. (B) Joint space trajectories for an exemplary learning participant of the extension group. The lines illustrate the mean trajectories for blocks B0, B2, B3, B4, B7 and B9. The initial and desired target configurations are indicated on the target solution manifold. (C) Actual visual noise added over the time course of learning split by experimental group. Error bars denote SEM across participants. (D) Percentage of rewarded trials over the time course of learning split by experimental group. Error bars denote SEM across participants.

To test whether the motor system learned a new wrist configuration in the manipulated direction along the manifold, i.e. more flexion or extension in the wrist compared to baseline, we analyzed Δ Wrist with a 2 (group) x 8 (experimental blocks B1-B8) mixed ANOVA (Fig 3A). We found no evidence for a main effect of time (*F*_*7*, *126*_ = 0.744; *p* = 0.635) or group (*F*_*1*, *18*_ = 1.896, *p* = 0.185). However, there was a significant interaction between group and time (*F*_*7*, *126*_ = 3.9, *p* < 0.001). Post-hoc analyses revealed significant group differences for the last two blocks in the expected direction (B7: *t*_*18*_ = 2.333, *p* = 0.015; B8: *t*_*18*_ = 2.259; *p* = 0.019; one-tailed). Furthermore, the effects seemed to be driven by subjects of the *extension group*, who exhibited a significant change in wrist behavior in the expected direction towards the end of the experiment (t-tests vs. 0; B7: *t*_*8*_ = 3.561; *p* = .004; B8: *t*_*8*_ = 3.159, *p* = 0.007; one-tailed), which is illustrated in Fig 3B for an exemplary participant. Interestingly, participants of both groups initially tended to extend their wrists with the onset of the noise gradient (t-test vs. 0; B1: *t*_*19*_ = 1.648, *p* = 0.058; one-tailed).

Exploring the data from another perspective, we also analyzed changes in the average visual noise that we injected (Fig 3C) with a 2 (group) x 8 (experimental blocks B1-B8) mixed ANOVA. Of interest, we found evidence for a main effect of time (*F*_*7*, *126*_ = 5.872; *p* < 0.001) but no effects for group (*F*_*1*, *18*_ = 2.637; *p* = 0.122) or group and time interaction (*F*_*7*, *126*_ = 0.223; *p* = 0.98). Indeed, post-hoc tests revealed that the motor system successfully reduced the injected variability by about 1 cm from B1 to B8 in both the *flexion group (t*_*10*_ = 2.403; *p* = 0.019; one-tailed) and the *extension group (t*_*8*_ = 2.344; *p* = 0.024; one-tailed). Lastly, we also analyzed participant’s average reward rates (Fig 3D) with a 2 (group) x 8 (experimental blocks B1-B8) mixed ANOVA. As expected, we found an effect of time (*F*_*7*, *126*_ = 7.352; *p* < 0.001). Further, we found no evidence for a main effect of group (*F*_*1*, *18*_ = 1.727; *p* = 0.205) or a group and time interaction (*F*_*7*, *126*_ = 0.567; *p* = 0.782). Post-hoc tests revealed that the percentage (%age) of rewarded trials increased from B1 to B8 in both the *flexion group (t*_*10*_ = 1.998; *p* = 0.037; one-tailed) and the *extension group (t*_*8*_ = 3.526; *p* = 0.004; one-tailed). Hence, even though a change in the behavioral variable of main interest, the wrist angle, was only found for the *extension group*, we demonstrated with the additional analyses that both groups re-gained more control over the cursor during training (Fig 3C), which resulted in higher reward rates for later blocks (Fig 3D).

There was no evidence for differences in learning towards the expected direction between unaware participants who had guessed the critical dimension correctly and those who had not (-0.17 ± 2.10°; *t*_*18*_ = -0.051, *p* = 0.960). To summarize, these results provide evidence that the motor system can adopt a new motor solution in joint space within a redundant task setting. The new movement strategy reduced the injected variability. Hence, the motor system learned this new arm configuration along the solution manifold without conscious awareness or explicit search process. However, in our context, we found evidence for this learning process only in the wrist *extension*, but not the wrist *flexion group*.

### No evidence for systematic change without reinforcement

One putative explanation for the asymmetry in learning between the extension and flexion groups is that participants may naturally increase the extension in their wrist over the course of the experiment, e.g. to reduce the amount of work that has to be contributed by the elbow. This hypothesis is in line with the trend for wrist extension that we observed across groups in the first learning block. Indeed, given the relatively short distance (15cm) between the start and the target position and the length of the wrist handle (16cm), participants could have substantially reduced the movement of the forearm by extending the wrist. In contrast, reaching movements that involve more wrist flexion lead to higher biomechanical costs [14,32,33] and likely larger signal-dependent noise [34] because they involve more elbow extension and thus a larger movement of the forearm. Therefore, wrist extension could hypothetically lead to an overall more efficient movement.

**Fig 4:**
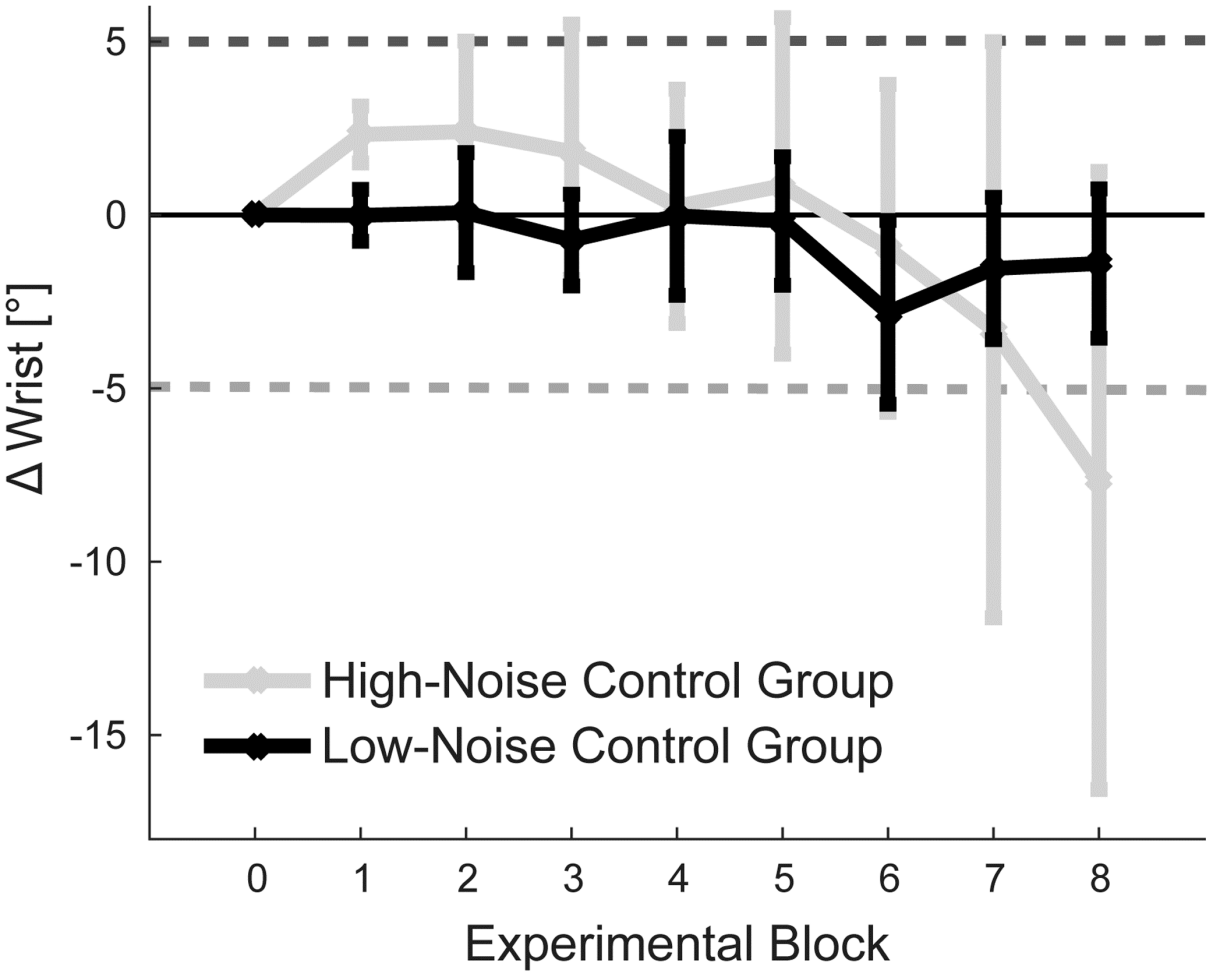
Behavior of control groups. Mean change in relative wrist angle over time with signed values (flexion negative, extension positive) for the low-noise control group and high-noise control group. Error bars denote SEM across participants. Horizontal lines indicate the learning goals for the two experimental groups depicted in Fig 3.

Thus, it is possible that a natural drift towards extension superimposed on the potentially symmetric learning curve for the two groups, making the learning appear asymmetric. To test for this hypothesis, we collected data from a *low-noise control group* in which participants were provided with veridical cursor feedback at the end of each trial, i.e. wrist joint configurations were not reinforced. This group, however, did not show any evidence for a systematic change of wrist angle at the end of the experiment (Fig 4, black line; B8: *t*_8_ = -0.650, *p* = 0.534).

Finally, it is also possible that the hypothesized drift only occurred in the presence of increased uncertainty in the environment, i.e. was induced by the noise added onto the visual endpoint feedback during the experimental learning blocks. This situation may for instance have induced exploration and hence may have allowed participants to discover the preferable movement strategy using wrist extension. To test for this hypothesis, we conducted the *high-noise control* experiment. In this condition, noise with a constant 2cm SD was added to the visual feedback of the cursor position, resulting in a similar manipulation throughout the experiment as for the experimental groups during early blocks (B1-B3) (injected noise high-noise control group: 2.53 ± 0.04cm; extension group: 2.06 ± 0.36cm; flexion group: 2.78 ± 0.36cm). However, also in this control group, wrist joint configurations were not reinforced. During debriefing, 2 of 7 participants from this control group indicated that the cursor feedback was manipulated and were thus excluded from the analysis.

Although the data suggest that participants extended initially (B1: 2.32 ± 0.82°, *t*_*4*_ = 2.823, *p* = 0.048), this strategy did not persevere. In contrast to our hypothesis, we found no evidence for a drift towards wrist extension such that by the end of the experiment movements did not differ significantly from baseline (B8: *t*_*4*_ = -0.859, *p* = 0.439). Altogether, we found no evidence for an underlying drift or optimization process towards wrist extension, independent of whether participants received veridical or noisy feedback. This alternative explanation for the asymmetric learning is therefore unlikely.

### Learning benefit of exploration is context dependent

Movement variability along task irrelevant dimensions has traditionally been regarded as motor noise and thus a movement feature that the motor system should decrease during a learning process. However, recent studies have suggested that exploration is a key element for successful learning in a redundant task setting [23,26]. In particular, Wu et al. [26] demonstrated that the variability in motor output during baseline along a task dimension that was first irrelevant but became relevant in the training phase, discriminated “good” from “bad” learners. Along this line, we hypothesized for our experiment that higher variability during baseline was associated with more learning in the expected direction along the solution manifold. We used the SD of Δ Wrist within the baseline block as a measure of variability that has been used as a proxy of exploration along the solution manifold [23,26].

**Fig 5:**
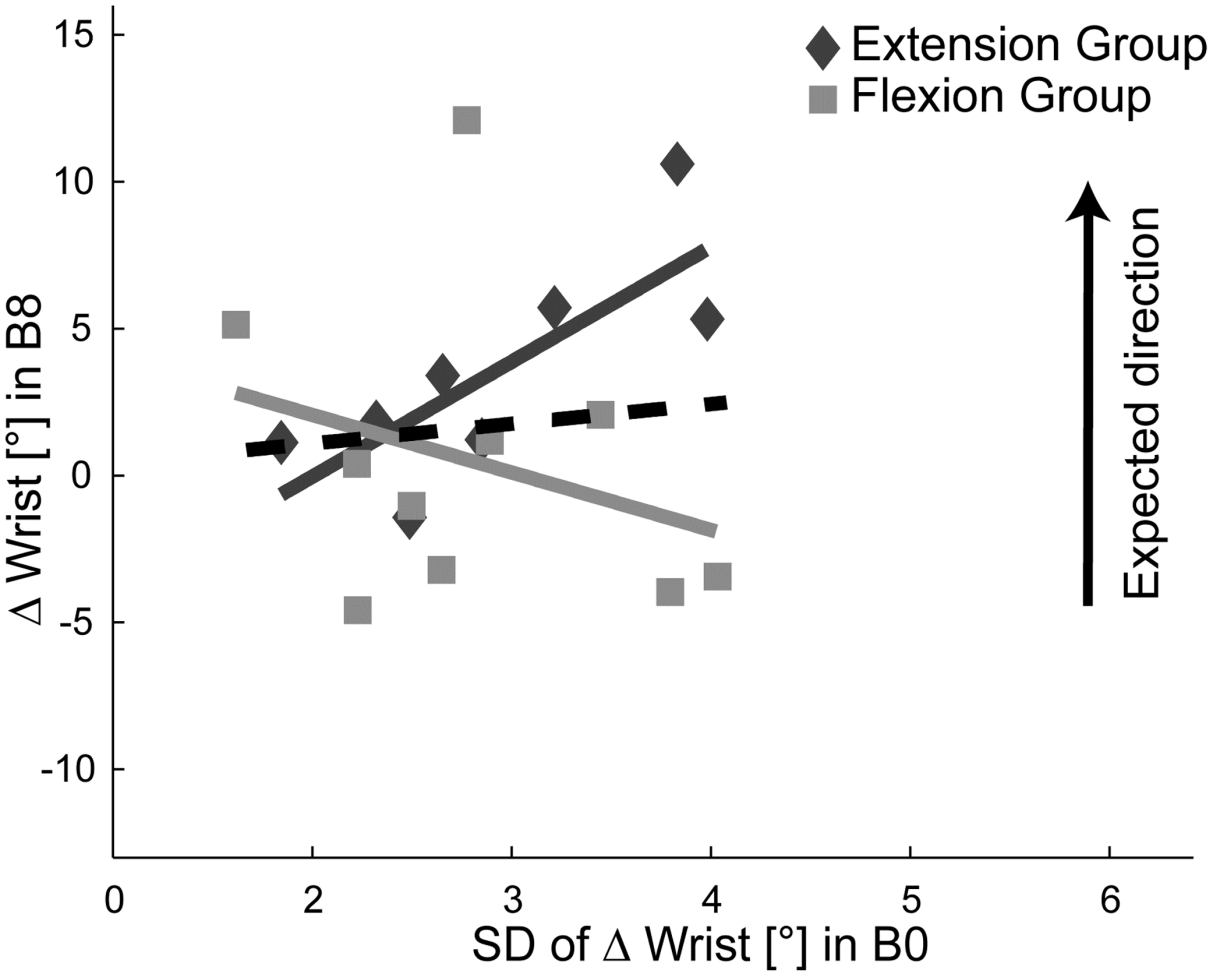
Variability and learning. Correlation between motor variability as a measure of exploratory behavior at baseline (B0) and change in wrist behavior at the end of the experiment (B8). Positive values were assigned to changes in the expected direction for each group. The grey lines visualize the regressions of the individual groups and the dashed black line the regression of the pooled data.

First, we asked whether the exploratory behavior of the pooled experimental participants during baseline correlated with the learning achieved at the last block (B8). For this specific analysis, we assigned positive values to changes in the expected direction for each group. In contrast to our expectations, no significant correlation was found (dashed black line in Fig 5; *r* = 0.205, *p* = 0.387).

When we split the analysis for the groups, we found a significant correlation between exploration and final wrist extension for the *extension group* (dark grey line in Fig 5; *r* = 0.78, *p* = 0.023), which supports the hypothesis of variability-driven learning success. However, we found no relationship for the *flexion group* (light grey line in Fig 5; *r* = -0.29, *p* = 0.42). If at all, higher exploration rather seemed related to slightly less learning success. This group difference between correlations was significant (*z* = 2.295; *p* = 0.022), suggesting that early exploration related to learning success only in the *extension group*. The control groups did not show any discernible relationship between baseline variability and later wrist extension (r = -0.37, *p* = 0.217) that could explain the observed tendency to flex the wrist.

Taken together, there was no evidence for a general positive relationship between baseline exploration and learning in the expected direction. We found this relationship only for the group that significantly changed their movement behavior in the desired direction, i.e. the *extension group*. This finding did not replicate in the three other groups that did not exhibit any change in movement strategy on the group level. We thus conclude that, with our experimental paradigm, the relationship between baseline exploration and learning outcome depended on the direction of the noise gradient, and that exploration did not lead to increased learning success per se.

## Discussion

To our knowledge, this study is the first to successfully induce implicit reinforcement learning in joint space of the arm. We investigated planar reaching movements in a redundant task setting that involved the coordination of shoulder, elbow and wrist joints [3]. The learning goal was to change the arm configuration at the endpoint towards larger wrist flexion or extension. The implicit teaching signal was the amount of added variability to the visual feedback of the end-effector position, or in other words, the controllability of the visual cursor. In most previous reinforcement learning studies, participants were made aware of the critical task dimension in the beginning of the experiment. In these studies, the manipulation of task success alone yielded learning [27,28,35]. In contrast, a recent study by Manley et al. [23] indicated that task success alone was not a sufficient teaching signal when participants were unaware of the critical dimension. However, the authors revealed that added extrinsic noise could serve as a successful teaching signal even in the absence of explicit awareness.

The present study replicated and extended the findings of Manley et al. [23]. While the previous study reduced task dimensionality by making the reaching direction task-irrelevant, we exploited here the natural redundancy of the arm in joint space for reaching a specified position in endpoint space. Thus, our new task comes closer to the real challenge of finding a favorable joint-space movement for a new motor skill. Moreover, in contrast to the previous study [23], the imposed noise gradient was determined by participants’ baseline behavior and remained constant across the whole experiment. Participants thus engaged in a redundant goal directed task for which only a subset of solutions led to optimal performance. However, as in the previous study, we found that added external noise can lead to slow reinforcement learning along the solution manifold. Taken together, our results therefore extend the previous findings to an ethologically more natural form of higher dimensional learning.

Surprisingly, we found a significant asymmetry in learning between the two groups. More specifically, only the *extension group* learned its intended new arm configuration. However, even though the *flexion group* did not show a net change of behavior into the desired direction compared to baseline (i.e. Δ Wrist), it reduced the injected noise by a similar amount as the *extension group*. Hence, both groups regained control over the cursor by a similar degree compared to the gradient onset. While we do not have a conclusive explanation for the observed asymmetry with respect to Δ Wrist, our additional results can rule out several potential reasons. First, participants might have naturally extended their wrist over the course of the experiment, as they learned to reduce the biomechanical costs in the other joints. Such an underlying drift superimposed on a symmetric learning effect could yield the observed patterns of results. However, a control group that received veridical cursor feedback at the end of each trial showed no systematic change of movement behavior. Secondly, the drift towards wrist extension might have occurred in response to increased task uncertainty. The injected variability could have induced exploration and thus facilitated that participants exploit wrist extension as the preferable movement strategy. However, a second control group, in which we increased the variability of the feedback also did not show a drift towards extension movements. These findings render an underlying optimization process or a natural drift an unlikely reason why only the *extension*, but not the *flexion group* had learned along the solution manifold.

The noise gradient was applied around the average solution chosen by the participants during the baseline phase. At the target, the wrist was on average at 165° (Fig 2A), i.e. 15° flexed relative to a neutral position of 180°. As the functional work range for daily activities ranges from 60° extension to 54° flexion [36] further flexion was biomechanically clearly possible and familiar to participants. Therefore, there were no *hard* biomechanical constraints in the way of optimization. Our results therefore suggest a natural bias in exploration, possibly induced by an asymmetric biomechanical cost function around the preferred baseline solution—with rapidly increasing costs for flexion. Interestingly, with the onset of added noise the *high-noise control group* (Fig 4), as well the extension and flexion groups (Fig 3A) showed an early tendency towards wrist extension. It is thus possible that the noise perturbation led to an initial wrist extension, as this direction of exploration was less costly than flexion. In our experimental context, only the *extension group* benefitted from initial wrist extension (i.e. could decrease the additional noise) and it was more likely to persevere with this strategy. While this admittedly post-hoc explanation remains to be tested in future experiments, our findings could suggest that biomechanical costs play an important role in shaping exploration and subsequent learning. Thus, it is possible that there are many such biomechanical biases that prevent the learner from finding the solution that minimizes task-relevant variability.

The main challenge for the motor system during implicit reinforcement learning is to identify the control variables it needs to change for maximizing reward. This task is also known as *structural credit assignment problem* in reinforcement learning [24,25]. It is inherent to any redundant task in which a low dimensional (teaching) signal in task space needs to be assigned to a higher dimensional execution space. When learning a new sport like tennis, for example, reward signals encode the success or failure of the entire motor program rather than of a single effector or movement component. It has been suggested that the motor system addresses this challenge by actively exploring different solutions along the manifold [7]. We thus investigated exploration as a possible driving force of learning. In contrast to a previous study [26], we did not find a correlation between baseline exploration and overall learning. However, a more detailed analysis of the experimental groups revealed such relationship for the *extension group* only. Thus, baseline variability explained inter-individual differences in learning only for the group that demonstrated robust learning across participants. Overall, the data supports the idea that the relationship between baseline variability and learning is dependent on the direction of the noise gradient. Indeed, another recent study suggests that task specific factors that affect variability determine learning rates (instead of variability per se) [37]. Lastly, we note that feedback about the endpoint error and reward were always presented together in our task and their respective contribution to learning rates can therefore not be disentangled. However, a similar study conducted by Manley et al. [23] contained conditions that allowed for manipulating task success and endpoint error in isolation. Their finding was that task success alone is not a sufficient, but necessary manipulation to induce learning if participants are unaware of the task critical dimension. Along this line, we suggest that the most likely driver of learning in joint space was the visual presentation of the endpoint error in the current study as well, but we cannot rule out that the binary feedback presentation also contributed to learning rates.

The phenomenon of learning new joint configurations along the *solution manifold* is also relevant to the process of stroke recovery. In general, improvements in post-stroke motor function can be achieved through the genuine recovery or through functional compensation [38–40]. After uni-lateral stroke, the control of distal joints such as the wrist is often impaired [41,42], and many stroke patients learn to compensate with proximal joints (e.g. the shoulder or trunk) for the impairment of distal joints (e.g. the wrist) [43]. Indeed, a recent simulation study found that unexpected joint coupling, rather than endpoint noise or muscle fatigue, contributes to impaired reaching performance after hemiparetic stroke [44]. Previous work suggests that this compensation may be driven by two learning mechanisms: Error-based learning will simply bring the movement endpoint back onto the *solution manifold*, and may therefore choose any solution in joint space that achieves this goal. The slower reinforcement learning will try to minimize the associated movement cost along the task relevant dimension [14,32]. Since the neural loss after stroke likely increases the movement cost for the affected joints (i.e. the wrist), the reward contingencies are such that they promote non-use of the affected limb, further preventing true recovery [45]. To promote genuine recovery of the affected body part, physical therapy needs to change the reward contingencies to promote movements of the affected joints [46]. Robot-mediated motor rehabilitation holds promising potential to promote neuroplasticity [47]. The use of a redundant robotic device allows to quantify functional compensation [48] and thus to exploit suitable learning mechanisms that promote post-stroke function. Our study demonstrates a training regimen that might yield success in this domain.

Compared to error-based learning, implicit reinforcement learning is a relatively slow process [11,23]. Also, in the current study, the amount that unaware participants learned was relatively low and took hundreds of trials to be achieved. While our current study did not assess retention of the motor memory, it has been shown that reinforcement learning leads to good retention of newly learned coordination patterns [35,49,50].

To summarize, this study showed that participants can learn new reaching strategies along the *solution manifold* to minimize variability without explicit knowledge of the critical task dimension. However, this process appears to be influenced and biased by biomechanical factors, which sometimes can prevent learning.
